# Additivity of resurgence and spontaneous recovery

**DOI:** 10.3758/s13420-026-00711-9

**Published:** 2026-02-25

**Authors:** Rodolfo Bernal-Gamboa, A. Matías Gámez, Christopher A. Podlesnik

**Affiliations:** 1 Universidad Nacional Autónoma de México, Mexico, Mexico; 2 Departamento de Psicología, Universidad de Córdoba, Córdoba, Spain; 3 Maimonides Institute for Biomedical Research of Córdoba (Spain), Reina Sofia University Hospital, Córdoba, Spain; 4 Department of Psychology, University of Florida, 945 Center Dr., Gainesville, FL 32611-2026 USA

**Keywords:** Additivity, Extinction, Humans, Resurgence, Spontaneous recovery

## Abstract

Resurgence and spontaneous recovery are two extensively studied behavioral phenomena in which previously extinguished operant behavior increases or reemerges. These findings are relevant to understanding flexibility in behavior but also relapse. Resurgence is the re-return of an extinguished target response (R1) when reinforcement conditions worsen for a more recently reinforced alternative response (R2), such as with extinction. Spontaneous recovery is the return of an eliminated R1 following time off from experiencing extinction conditions, such as with a retention interval. The present experiment examined whether combining tests for resurgence (removing R2 reinforcement) and spontaneous recovery (retention interval) produced greater increases in R1 than either test in isolation. University students played a computer video game and learned to perform R1 during Phase 1. During Phase 2, R1 was placed on extinction while simultaneously performing R2 was reinforced (Ext + DRA). Finally, the four groups of participants were tested for (1) neither resurgence or spontaneous recovery, (2) only resurgence, (3) only spontaneous recovery, or (4) both resurgence and spontaneous recovery. Increases in R1 were additive, with combined testing producing significantly greater responding that either of the two tests in isolation. We discuss theoretical issues as well as the implications of our findings for the treatment and relapse of challenging behavior.

Research on the effects of extinction reveals the elimination of reinforcers contingent upon a previously maintained operant response does not eliminate prior learning. Retention of learning has been demonstrated by several environmental changes that increase responding during extinction without additional training (see Bouton et al., 2021; McConnell & Miller, 2014; Wathen & Podlesnik, 2018, for reviews). Examples include *renewal* resulting from changes in the environmental context (e.g., Bernal-Gamboa et al., 2017, 2020) and *reinstatement* resulting from re-presenting the previous reinforcer noncontingently (e.g., Miranda-Dukoski et al., 2016). These findings reveal responding eliminated by extinction reflect changes in performance of operant behavior. Further, the capacity of operant behavior to increase or reemerge following extinction reflects a form of behavioral flexibility relevant to adaptation and problem solving (e.g., Williams & St. Peter, 2019) but also the potential for relapse despite successful intervention (e.g., Bouton, 2019).

Another example in which extinguished behavior increases or re-emerges is *spontaneous recovery*. A retention interval providing time off from exposure to extinction results in an increase in responding with re-exposure to extinction contingencies (e.g., Bernal-Gamboa et al., 2017; Rescorla, 1996, 1997). In one experiment with university students, Gámez and Bernal-Gamboa (2019) had participants shooting (mouse clicks) and destroying enemy vehicles according to a variable-interval (VI) 2-s schedule during Phase 1. After the shooting response was acquired above 100 responses per min, Phase 2 arranged extinction and shooting declined below 20 responses per min. After a retention interval of 48 h, spontaneous recovery was demonstrated by shooting increasing above 40 responses per min. Findings of spontaneous recovery reveal sustained effects of acquisition learning despite decrements in responding from extinction (see also Mason et al., 2023; Thrailkill et al., 2018). They further suggest interventions designed to decrease clinically relevant challenging behavior (self-injury, aggression) with extinction could be susceptible to relapse (e.g., Lerman et al., 1999).

A related phenomenon also demonstrating extinction does not eliminate prior learning is *resurgence*. Resurgence has been defined as a return of a previously reinforced and extinguished response, R1, when conditions worsen for a more recently reinforced response, R2 (see Lattal et al., 2017). Worsening conditions for R2 have included extinction or any decrease in the reinforcer rate or magnitude contingent upon R2 (see Podlesnik et al., 2022, for a systematic review). For an example with adult humans, Ritchey et al. (2021) reinforced on-screen button presses as R1 with points exchangeable for money during Phase 1, R1 rates exceeded 100 responses per min. During Phase 2, R1 was extinguished to zero rates while a different button press, R2, was reinforced with points and exceeded 100 responses per min. During Phase 3, extinction of R2 was introduced, resulting in decreases in R2 and increases in R1 to approximately 40 responses per min. These increases in R1 demonstrated resurgence due to worsening of R2 conditions with extinction and further reveal sustained operant learning throughout extinction (see also Kestner et al., 2018; Thrailkill et al., 2019). Given the use of differential reinforcement of alternative behavior (DRA) during many treatments for clinically relevant behavior (see Greer et al., 2016), resurgence of R1 implies the possibility of a return of clinically relevant challenging behavior when reinforcement conditions worsen during DRA treatment. Increases in clinically relevant behavior can result from treatment-integrity errors or planned schedule thinning of the DRA treatment (see Briggs et al., 2018; Muething et al., 2021; Volkert et al., 2009).

Several studies have shown those events producing a return of extinguished behavior in isolation, such as resurgence and spontaneous recovery, produce even greater increases in responding when tested in combination (see King et al., 2024, for a systematic review). For example, Ritchey et al. (2022a, 2022b) evaluated both resurgence and renewal in adult humans pressing onscreen buttons maintained by points. Across three phases, they arranged four groups experiencing training on one context (A), DRA and extinction in a novel context (B), and either a return to context A (ABA) or remaining in context B (ABB). Specifically, they arranged tests during Phase 3 for (1) Group ABA − as ABA context changes (renewal) and removal of alternative reinforcement (resurgence), (2) Group ABA + as ABA context changes (renewal) while maintaining alternative reinforcement (no resurgence), (3) Group ABB − as ABB context changes (no renewal) and removal of alternative reinforcement (resurgence), and (4) Group ABB + as ABB context changes (no renewal) while maintaining alternative reinforcement (no resurgence). The ABA − group experiencing both a return to Context A and removal of R2 reinforcers exhibited greater R1 levels during testing than the other three groups (see also Trask & Bouton, 2016). Moreover, Ritchey et al. found R1 levels exceeded the sum of R1 responding from both of the isolated ABA + and ABB − groups, indicating a super-additive effect of changing context and removing R2 reinforcers. In terms of DRA treatment, the effects of context changes and removal of R2 reinforcers could synergize and exacerbate increases in challenging behavior (see King et al., 2024, for a discussion).

The present study extended previous research demonstrating isolated findings of spontaneous recovery (e.g., Gámez & Bernal-Gamboa, 2019; Zeiler, 1971) and resurgence (e.g., Craig et al., 2020; Leitenberg et al., 1970). With university students as participants, we tested for the first time whether the inclusion of (1) a 48-h retention interval before Phase-3 extinction testing and (2) removal of R2 reinforcers during Phase 3 resulted in greater returns of R1 than groups experiencing either manipulation in isolation. We also included a control group experiencing neither manipulation. We predicted greatest increases in R1 in the group with combined manipulations, similar R1 increases for groups experiencing the manipulations in isolation, and no R1 increases for the group experiencing no change between Phases 2 and 3. If R1 increases were greater in the combined group, we evaluated whether those increases were greater than the sum of groups experiencing isolated tests, i.e., *super-additive*; Ritchey et al., 2022a, 2022b), equivalent to the sum of isolated groups (i.e., *additive*; Trask & Bouton, 2016), or less than the sum of isolated groups, which could either be *subadditive* or demonstrate no increase above either group in isolation (e.g., Sweeney & Shahan, 2015).

## Method

### Participants

We calculated the sample size for a large effect size of 0.14 (Cohen’s *f* of 0.40). For instance, Gámez and Bernal-Gamboa (2019) found a η_p_^2^ of 0.30 for a combined spontaneous recovery + renewal effect. This was obtained based on a 4 × 2 × 2 mixed analysis of variance (ANOVA), with 2 Phase (2 measurements: Last extinction trial of Phase 2, Phase 3), and 2 Response (2 measurements: R1, R2) as within-subjects factors, and 4 Group as a between-subjects factor (combined, spontaneous recovery, resurgence, no reoccurrence), using G*Power 3.1.9.7 (Faul et al., 2007). We used an alpha of 0.05, a power of .80, a correlation among repeated measures of 0.5, and a nonsphericity correction of 1. The results of the sample size calculation indicated that 16 participants were needed to achieve a power of 0.83. No volunteer who showed up was rejected, resulting in 80 participants (20 per group). They were students from the National University of Mexico (Mexico) and participated in the present experiment in exchange for course credit (59 women; *M*_age_ = 20.4 years old). They had no previous experience with this task. All students participated voluntarily and gave their informed consent before beginning the experiment, being free to discontinue the task at any point, though none of them did.

### Apparatus and stimuli

**Fig. 1:**
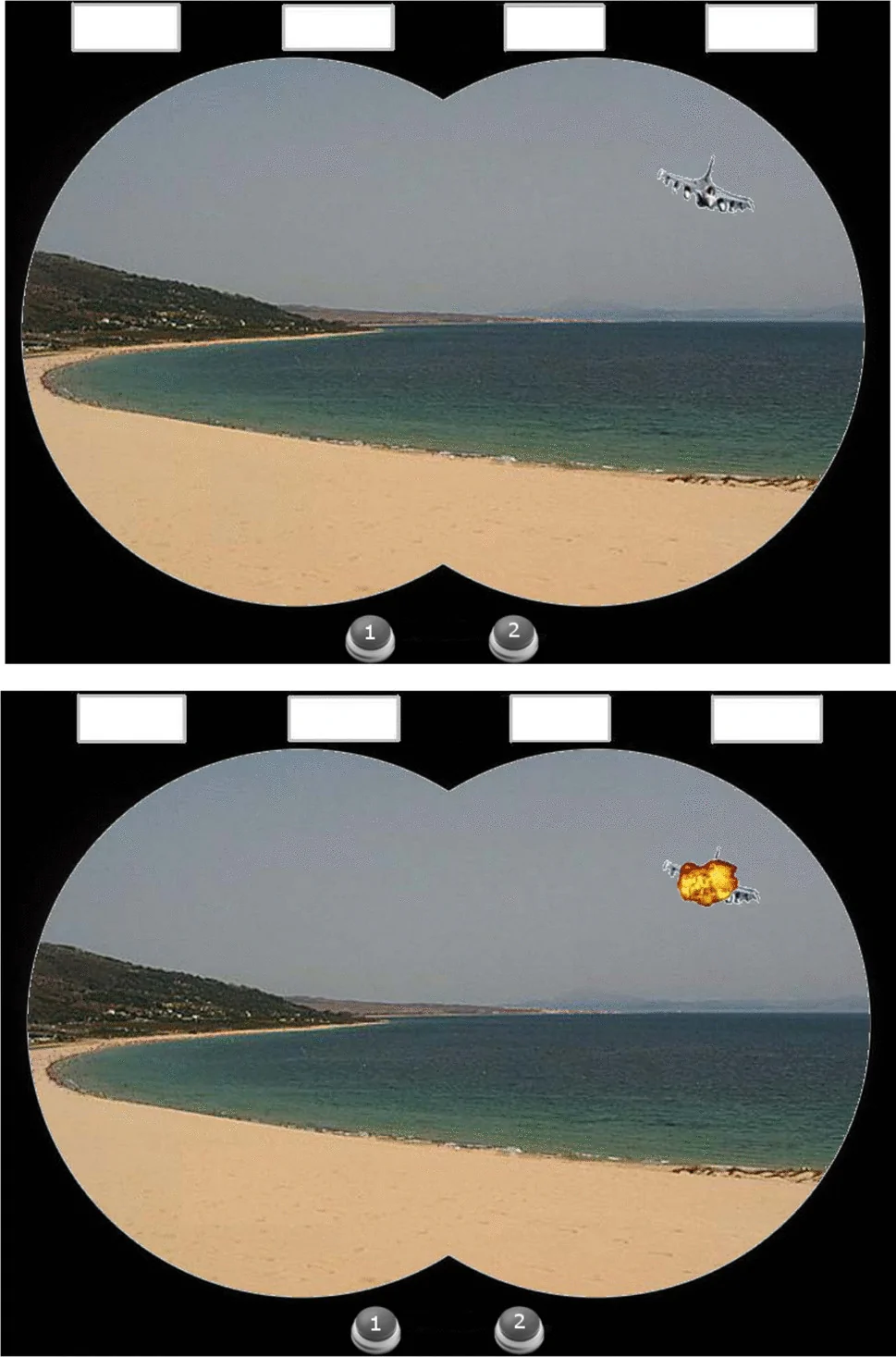
Screenshot of computer interface. Example of a screen with two keys to click on (top) and an example of a screen with reinforcement (bottom). (Color figure online)

Participants were trained individually, in adjacent PCs separated by fixed partitions. The procedure was implemented using the SuperLab Pro (Cedrus Corporation) software. Participants played a computer game in which they had to defend Andalusia from air attacks. The main screen represented a viewer, simulating a participant’s view from a hypothetical bunker. A scene of Tarifa (a beach in Andalusia) was presented within the viewing area. A plane was presented in the sky, at the top right area of the screen and could appear in one of two different positions within its respective area to give the impression of movement to the participant. The operant response consisted of clicking on either of the two keys (R1 and R2, counterbalanced) at the bottom center of the screen. The destruction of the plane was used as the outcome (see Fig. 1).

### Procedure

The present experimental protocol was conducted under strict agreement of the guidelines established by the Ethical Committee of the Faculty of Psychology of the National University of Mexico. In addition, it was approved by the Human Ethics Committee at the University of Cordoba under protocol number CEIH-21–24. The instructions and all necessary information were presented in the participants’ native language (Spanish) on the computer screen. Participants interacted with the computer using the left button of the mouse. Instructions were presented in three screens using a black Times New Roman 26 bold font against a light-yellow background, to emulate the appearance of an old document. To advance through the instructions screens, participants had to click on a button labeled “next,” placed on the bottom right corner of the screen. Each participant was initially asked to read the following instructions:

Screen 1: “Andalusia is under attack. Different parts of Andalusia are being assaulted by air. You are placed in the only bunker able to face the attackers. Use the mouse to launch missiles at the targets. Your goal is to destroy the attackers before they take over Andalusia.”

Screen 2: “The monitor represents the bunker’s viewer, in which the different attackers you should face will appear. To shoot, click the left mouse button while the pointer is on the keys that will appear on the bottom of the screen. Your technology and weapons are older than theirs, so you will need to shoot several times to destroy them.”

Screen 3: “The battle begins! You have to destroy the planes before they take over the Andalusian coast. We are in your hands! GOOD LUCK!”

In each trial, the plane was presented. Giving the appropriate response (i.e., clicking on the 1 key) was reinforced with the destruction of the attacker (outcome) on a VI 2-s schedule in which the availability of reinforcement varied randomly between 1 and 3 s—VI 2-s schedules have been used extensively in studies with humans examining related phenomena (e.g., Gámez & Bernal-Gamboa, 2019; Montague et al., 2025). The trial ended only after the participant gave the correct response and, hence, the enemy was destroyed. Each trial began immediately after the previous one; that is, there was no intertrial interval (ITI). Thus, the present task can be considered as a trial-based approach, which is a procedure quite commonly used in studies with humans examining related phenomena (e.g., Baiamonte & Gámez, 2025; Vila et al., 2022). Table 1 shows the experimental design for the four groups and three phases.

**Table 1:** Experimental design

Group	Phase 1	Phase 2	Retention interval	Phase 3
Combined	12: R1 +	24: R1 − , R2 +	48 h	4: R1 − , R2 −
Spontaneous recovery	12: R1 +	24: R1 − , R2 +	48 h	4: R1 − , R2 +
Resurgence	12: R1 +	24: R1 − , R2 +	0 h	4: R1 − , R2 −
No recurrence	12: R1 +	24: R1 − , R2 +	0 h	4: R1 − , R2 +

“R1” and “R2” stands for clicking on two on-screen buttons (left and right), counterbalanced. “ + ” means that performing the operant response was reinforced. “ − ” means that performing the operant response was not reinforced. The numbers before the letters refer to the number of trials conducted in each phase

#### Phase 1

A screen displaying the message “Your detachment has been posted to Tarifa” was presented for 2 s before starting training. All participants received 12 training trials with the attacker. Only one on-screen key was available (e.g., key 1), and responses were reinforced according to a VI 2-s schedule.

#### Phase 2

After Phase 1, a screen displaying the message “Your detachment has been posted to Tarifa” was presented for 2 s before starting this phase for all four groups. All participants received 24 trials with the plane displayed onscreen. More trials were used in this Phase than in Phase 1, consistent with previous reports using a task similar to the one presented here (see Gámez & Bernal-Gamboa, 2019). This is mainly because extinction of the instrumental response in this task is more demanding than acquisition, most likely because the response is very easy to perform. Two on-screen keys were available (1 and 2). Responses were never reinforced for clicking on key 1 presented on the previous phase, while clicking on the alternative key 2 was reinforced on a VI 2-s schedule.

#### Phase 3

For all participants, this phase started with the sentence: “Your detachment has been posted to Tarifa,” which was presented for 2 s. After that, all participants received four test trials with the plane displayed onscreen. Two on-screen keys were available (1 and 2). Each trial lasted 4 s. This is the duration of a trial during the acquisition phase, provided that the participant responds when the enemy appears. Participants in the No reoccurrence and Resurgence groups received this phase immediately after Phase 2, whereas the test was conducted two days after Phase 2 for participants in the Spontaneous recovery and Combined groups. In addition, in the No reoccurrence and Spontaneous recovery groups, testing involved the same arrangement of Phase 2 (R2 was reinforced, while R1 was not); while in the Resurgence alone and Combined groups, no responses were reinforced.

### Dependent variable and statistical analysis

The total number of mouse clicks on the on-screen keys were recorded and presented as responses per minute. Although our procedure is a discrete-trial design—in which the number of responses per trial is commonly used as the dependent variable—the use of responses per minute is also common in the literature (e.g., Gámez & Bernal-Gamboa, 2018; Podlesnik et al., 2025) and additionally allows for the comparison of response rates across phases. The data were analysed using mixed ANOVAs and appropriate *t* tests. When necessary, the Greenhouse–Geisser correction was applied to account for violations of the sphericity assumption. Simple effects were conducted with *t* tests or one-way ANOVAs, using in this case corrections when appropriate (HSD Tukey). Effect sizes for the overall ANOVA are reported as partial eta-squared (η_p_^2^), and Cohen’s *d* is reported for the *t* tests, and 95% confidence intervals for the effect sizes were reported for each analysis relevant to our hypotheses (bracketed). The rejection criterion was set at *p* < .05.

## Results

### Phase 1

**Fig. 2:**
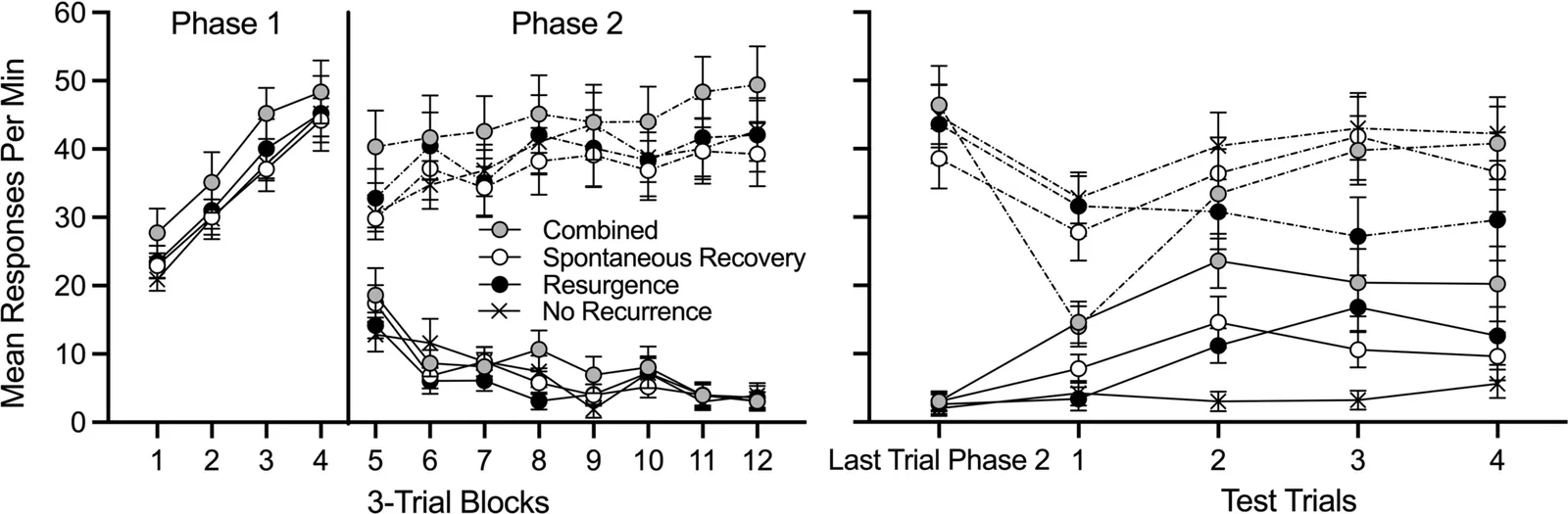
Response rates across three phases. On the left and central panel, mean R1 and R2 response rates across Phases 1 and 2 are displayed across three-trial blocks. On the right panel, mean R1 and R2 response rates are displayed across the last trial of Phase 2 and the four Phase 3 trials. Solid lines represent R1 and dashed lines represent R2. Error bars are SEM.\

The left panel of Fig. 2 displays the mean responses per minute for R1 across the four participant groups during Phase 1. A mixed 4 (Group) × 4 (Block) ANOVA conducted on the acquisition data confirmed that only the main effect of Block was significant, *F*(2.06, 156.71) = 103.29, *p* < .001, ηₚ2 = .58, whereas neither the main effect of Group nor the Group × Block interaction reached significance, *F*s < 1. This indicates that, regardless of group, the R1 response rate increased progressively across trial blocks.

### Phase 2

During Phase 2 (central panel of Fig. 2), R1 responding decreased while R2 responding increased in a similar manner across all four groups. A mixed 4 (Group) × 2 (Response) × 8 (Block) ANOVA conducted on these data revealed a significant main effect of Response and a significant Response × Block interaction, *F*(1, 76) = 176.98, *p* < .001, ηₚ2 = .70, and *F*(4.96, 377.21) = 29.25, *p* < .001, ηₚ2 = .28, respectively. All other main effects and interactions were non-significant, largest *F* = 1.38, *p* = .21. Analysis of the interaction showed that the simple effects of Response were significant at every block, smallest *t*(79) =  − 7.79, *p* < .001, *d* = .87. Additionally, the simple effect of Block was significant for both R1, *F*(4.86, 383.81) = 20.90, *p* < .001, ηₚ2 = .21, and R2, *F*(3.89, 307.48) = 14.83, *p* < .001, η2ₚ = .16. In other words, as Phase 2 progresses, there was a decrease in R1 and an increase in R2, irrespective of the group.

### Phase 2 and 3 (test)

The right panel of Fig. 2 displays the R1 and R2 response rates for the four groups during the last trial of Phase 2 and the four Phase 3 trials. To evaluate our initial hypotheses, we compared the response rates across the four groups from the last trial of Phase 2 to the first trial of Phase 3. A mixed 4 (Group) × 2 (Response) × 2 (Phase) ANOVA revealed a main effect of Response, *F*(1, 76) = 157,11, *p* < .001, ηₚ2 = .67, and a main effect of Phase *F*(1, 76) = 42.38, *p* < .001, ηₚ2 = .36. 42. Moreover, two-way interactions Group × Phase and Response × Phase reached significance, *F*(3, 76) = 2.94, *p* = .038, ηₚ2 = .10, and *F*(3, 76) = 2.94, *p* = .038, ηₚ2 = .10, respectively. Finally, the three-way interaction was significant as well, *F*(3, 76) = 8.62, *p* < .001, ηₚ2 = .25.

### Analysis of R1

As a follow-up to the three-way interaction analysis, we first compared the response rates across Phases 2 and 3 for R1, the primary dependent variable of interest for our hypotheses. A mixed 4 (Group) × 2 (Phase) ANOVA using only R1 data revealed a main effect of Group, *F*(3, 76) = 3.21, *p* = .028, ηₚ2 = .11, and of Phase, *F*(1, 76) = 21.63, *p* < .001, ηₚ2 = .22. Most importantly, the Group × Phase interaction was also significant, *F*(3, 76) = 5.29, *p* = .002, ηₚ2 = .17. The analysis of this interaction revealed a significant simple effect of Group in the first trial of Phase 3, *F*(3, 79) = 5.23, *p* = .002, η2 = .17 [.03; .30], but not during the last trial of Phase 2, *F* < 1. Post hoc comparisons in Phase 3 revealed higher R1 responding only in the Combined group compared to the No Recurrence group, *p* = .008. So, these data do not support the presence of both spontaneous recovery and resurgence effects. Furthermore, the simple effect of Phase was significant in the Combined group, *t*(19) =  − 3.47, *p* = .001, *d* =  − .77 [− 1.27; − .26], in the Spontaneous Recovery group, *t*(19) =  − 2.40, *p* = .013, *d* =  − .54 [− 1.00; − .06], and in the No Recurrence group, *t*(19) =  − 1.99, *p* = .030, *d* =  − .44 [− .90; .02]. In contrast, the Resurgence group did not show a significant difference, *t*(19) < 1, *p* = .21. It is likely that a single test trial was not sufficient to capture the resurgence effect, as the participant experiences the absence of reinforcement for R2 within that same trial. As can be seen in the right panel of Fig. 2, R1 increases in the second trial of Phase 3 in the Spontaneous Recovery, Resurgence, and Combined groups, whereas in the No Recurrence group it remains constant. Therefore, to assess the presence of the relapse effects under study, it seems appropriate to compare the rate of R1 in the last trial of Phase 2 with the average across Phase 3.

A mixed 4 (Group) × 2 (Phase) ANOVA revealed a main effect of Group, *F*(3, 76) = 5.48, *p* = .002, ηₚ2 = .18, and of Phase, *F*(1, 76) = 44.31, *p* < .001, ηₚ2 = .37. Most importantly, the Group × Phase interaction was also significant, *F*(3, 76) = 9.82, *p* < .001, ηₚ2 = .28. The analysis of this interaction revealed a significant simple effect of Phase in the Combined group, *t*(19) =  − 4.82, *p* < .001, *d* =  − 1.07 [− 1.62; − .51], in the Spontaneous Recovery group, *t*(19) =  − 4.21, *p* < .001, *d* =  − .94 [− 1.46; − .40], and in the Resurgence group, *t*(19) =  − 5.10, *p* < .001, *d* =  − 1.14 [− 1.69; − .56], but not in the No Recurrence group, *t*(19) < 1, *p* = .31. As expected, a 48-h delay between Phases 2 and 3 (Spontaneous Recovery group), the omission of reinforcement following R2 in Phase 3 (Resurgence group), or the combination of both manipulations (Combined group), led to a recovery of R1 in Phase 3. Furthermore, the simple effect of Group was significant during Phase 3, *F*(3, 79) = 11.64, *p* < .001, η2 = .31 [.13; .44], but not during the last trial of Phase 2, *F* < 1. Post hoc comparisons in Phase 3 revealed higher R1 responding in the Spontaneous Recovery and Resurgence groups compared to the No Recurrence group, *p* = .007 and *p* = .010, respectively. These between-group comparisons further support the presence of both spontaneous recovery and resurgence effects. Moreover, significant differences were also observed between the Combined group and each of the other three groups: Spontaneous Recovery (*p* = .01), Resurgence (*p* = .02), and No Recurrence (*p* < .001). That is, R1 responding was significantly greater in the Combined group than in any other group.

**Fig. 3:**
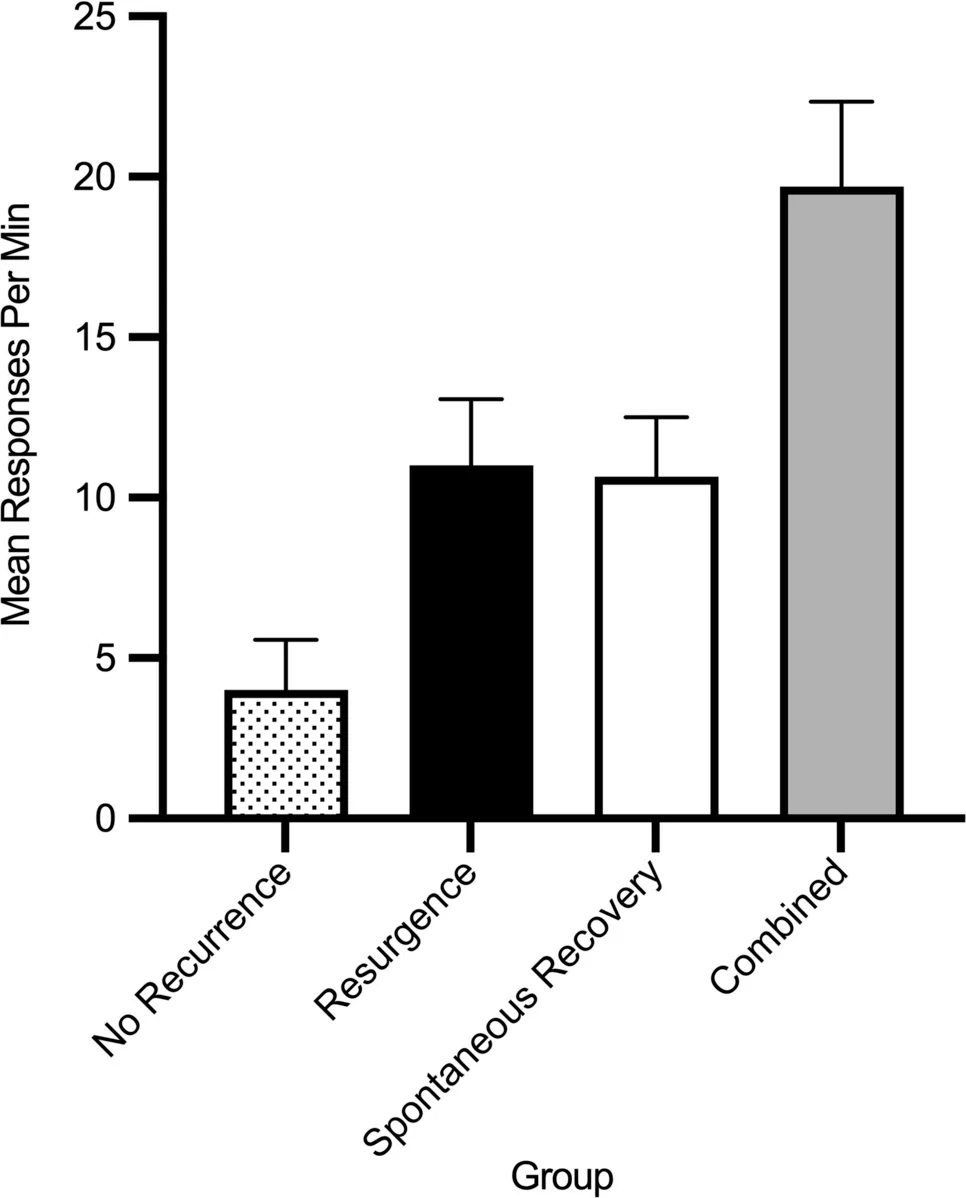
Response rates for R1 during Phase 3. Mean response rates for R1 are displayed for the four Phase3 trials. Error bars are *SEM*

Finally, Fig. 3 shows mean response rates across trials during Phase 3. To determine whether the combined effect of spontaneous recovery and resurgence was additive (equal to the sum of these effects in isolation) or super-additive (greater than the sum), we compared the Combined group (Mean = 19.7) to the sum of R1 responses in the Spontaneous Recovery and Resurgence groups (Mean = 21.6). This comparison revealed no significant difference, *t*(38) < 1, *p* = .32. That is, there is an additive effect of combining spontaneous recovery and resurgence.

### Analysis of R2

Continuing with the analysis of the three-way interaction, we compared the response rates across phases 2 and 3 for R2. A mixed 4 (Group) × 2 (Phase) ANOVA using only R2 data revealed a main effect of Phase, *F*(1, 76) = 73.56, *p* < .001, ηₚ2 = .49, but not of Group, *F* < 1. The Group × Phase interaction was significant as well, *F*(3, 76) = 7.07, *p* < .001, ηₚ2ₚ = .22. The analysis of the interaction revealed a significant simple effect of Phase in the Combined, *t*(19) = 5.51, *p* < .001, *d* = 1.23, the Spontaneous Recovery, *t*(19) = 3.20, *p* = .002, *d* = .72, the Resurgence, *t*(19) = 4.48, *p* < .001, *d* = 1.00, and the No Recurrence groups, *t*(19) = 4.15, *p* < .001, *d* = .93. That is, the response rate was significantly lower in the first trial of Phase 3 than at the end of Phase 2. Moreover, the simple effect of Group reached statistical significance for phase 3, *F*(3, 79) = 5.01, *p* = .003, η2 = .16, but not for phase 2, *F* < 1.

## Discussion

We explored the impact of combining tests for resurgence and spontaneous recovery in an operant task with humans. The reappearance of the extinguished response produced by removing R2 reinforcement (resurgence) and by including a 48-h retention interval (spontaneous recovery) are in line with previous findings in humans (e.g., Gámez & Bernal-Gamboa, 2019; Podlesnik et al., 2020) and nonhuman animals (e.g., Epstein, 1983; Rescorla, 1997). This also was to our knowledge the first demonstration of spontaneous recovery of R1 with differential reinforcement and extinction contingencies in place. Relatedly, Mazur (1995, 1996) observed spontaneous recovery of previous choice patterns in pigeons under concurrent reinforcement schedules. Furthermore, the most important finding of the present experiment was to provide for the first time data showing an additive effect when combining tests for resurgence and spontaneous recovery. We found greater increases in R1 during the combined test than during either test in isolation.

The impact on recurrence of combining resurgence and spontaneous recovery tests is consistent with other studies reporting higher levels of response recovery when combining resurgence and renewal tests (e.g., Alessandri & Cançado, 2020; Kincaid et al., 2015; Podlesnik et al., 2019; Ritchey et al., 2022a, 2022b; but see Sweeney & Shahan, 2015), resurgence and reinstatement tests (e.g., Liggett et al., 2018; but see Bouton & Trask, 2016); as well as combining tests of spontaneous recovery and renewal (e.g., Gámez & Bernal-Gamboa, 2019; Vila et al., 2002). When combining tests of spontaneous recovery and resurgence in the present experiment, we found response rates were significantly higher than when testing for spontaneous recovery and resurgence in isolation. It is worth noting that the additive effect likely is a function of the specific variables arranged in the present experiment. Arranging different R1 and R2 reinforcer rates or different durations of retention interval could have produced outcomes other than additivity. Further, different durations of testing could influence such outcomes, as we observed no reliable resurgence or spontaneous recovery during the first test trials for these groups (see Podlesnik & Kelley, 2014, for related findings and discussion). Parametric manipulations of these types of variables in combination have yet to be studied but would provide important insights into how such variables influence the increase or re-emergence of operant behavior.

Most studies examining resurgence and spontaneous recovery of operant behavior have arranged free-operant conditions of reinforcement (see Podlesnik et al., 2022, for a systematic review). In contrast, responses in the present study could only be produced during the presence of the enemy aircrafts, which were presented as discrete trials (see also Fujimaki et al., 2024; Gámez & Bernal-Gamboa, 2019; Liddon et al., 2017; Ritchey et al., 2022a, 2022b). We also examined only brief testing of resurgence and spontaneous recovery with across four test trials, which precluded us from evaluating how persistent R1 or R2 were across the different testing conditions. The influence of both these factors could be examined in future studies. Re-presentations of the enemy aircrafts during discrete trials were the discriminative stimuli setting the occasion for responding. As a result, aircraft presentation could increase the persistence of responding overall compared with enemy aircraft that were constantly available (see Fujimaki et al., 2024, for a discussion), as would be the case with free-operant procedures (see Ritchey et al., 2025).

The aforementioned findings are consistent with a theoretical perspective suggesting that, despite the methodological differences between resurgence, spontaneous recovery, reinstatement, and renewal, all these behavioral phenomena share similar mechanisms (e.g., Bouton, 2017, 2019). Rather than unlearning, the mechanism proposed is the decrease in responding observed during extinction reflects inhibitory learning of R1 and little generalization of R1 to contexts other than extinction (e.g., Trask et al., 2017). Therefore, conducting the test under conditions or contexts other than extinction will favor the return of R1. Although only renewal involves changes in physical contexts, this perspective has proposed that other stimuli can play the role of context, such as the reinforcement of an alternative response, delivering the outcome after extinction, or cues correlated with the passage of time (e.g., Bouton, 2010, 2019). One clear implication of the above-mentioned assumption is that combining contexts should have a greater effect on response recovery than the separate effect of one contextual change (e.g., Bouton, 1993). Consistent with this interpretation, the additive increases during Phase 3 demonstrated in the Combined group can be interpreted as a joint effect of removing alternative reinforcement and changing temporal context. The combination of manipulations would be consistent with a greater overall reduction in extinction’s inhibitory effects compared with either in isolation.

The results presented here may be relevant to clinical psychologists. It is well known the return of extinguished responses during resurgence and spontaneous recovery share certain parallels with the relapse of clinically relevant behavior observed after therapeutic interventions (e.g., Kimball et al., 2023; Wathen & Podlesnik, 2018), including avoidance of conditional fear responses in individuals with phobias and challenging behavior displayed by individuals with developmental disabilities. In the case of the treatment of challenging behavior (e.g., self-injury, aggression), a common treatment involves placing problematic behavior on extinction and providing reinforcement following a functionally equivalent communication response (i.e., functional communication training; e.g., Muething et al., 2021; Saini et al., 2018). The present findings suggest treatment disruptions of time off from treatment (e.g., weekends, vacation) in combination with treatment-fidelity errors involving the failure to reinforce communication responses could exacerbate the increase or re-emergence of challenging behavior compared to either treatment disruption in isolation (see King et al., 2024, for a related discussion). Preclinical research through laboratory models related to the present findings could be designed to further develop our understanding of how these treatment disruptions contribute to such lapses in clinically relevant challenging behavior and to develop methods to mitigate those effects (see King et al., 2026, for related findings).

## Data Availability

Experiment data could be available under request.

## Declarations

### Open practices statement

None of the data or materials for the experiments reported here are available, and none of the experiments were preregistered. However, experiment data could be available under request.
